# Bevacizumab-induced immune thrombocytopenia in an ovarian cancer patient with mixed connective tissue disease: case report and literature review

**DOI:** 10.3389/fimmu.2024.1382964

**Published:** 2024-06-05

**Authors:** Yunting Zhang, Fanchun Yang, Jining Wang, Hui Fu, Fuming Shen, Jie Liu, Dongjie Li

**Affiliations:** ^1^ Department of Pharmacy, Shanghai Tenth People’s Hospital, Tongji University School of Medicine, Shanghai, China; ^2^ Department of Obstetrics and Gynecology, Shanghai Tenth People’s Hospital, School of Medicine, Tongji University School of Medicine, Shanghai, China

**Keywords:** drug-induced immune thrombocytopenia, bevacizumab, autoimmune diseases, ovarian cancer, mixed connective tissue disease, case report

## Abstract

Drug-induced immune thrombocytopenia is an adverse reaction marked by accelerated destruction of blood platelets. In cancer therapy, thrombocytopenia has many other causes including bone marrow suppression induced by chemotherapeutic agents, infection, and progression of cancer; drug-induced thrombocytopenia can easily be misdiagnosed or overlooked. Here, we present a case of an ovarian cancer patient with a history of mixed connective tissue disease who underwent surgery followed by treatment with paclitaxel, cisplatin, and bevacizumab. The patient developed acute isolated thrombocytopenia after the sixth cycle. Serum antiplatelet antibody testing revealed antibodies against glycoprotein IIb. After we analyzed the whole therapeutic process of this patient, drug-induced immune thrombocytopenia was assumed, and bevacizumab was conjectured as the most probable drug. Thrombocytopenia was ultimately successfully managed using recombinant human thrombopoietin, prednisone, and recombinant human interleukin-11. We provide a summary of existing literature on immune thrombocytopenia induced by bevacizumab and discuss related mechanisms and triggers for drug-induced immune thrombocytopenia. The present case underscores the potential of bevacizumab to induce immune-mediated thrombocytopenia, emphasizing the need for heightened vigilance towards autoimmune diseases or an autoimmune-activated state as plausible triggers for rare drug-induced immune thrombocytopenia in cancer therapy.

## Introduction

Thrombocytopenia is a frequent hematological adverse reaction of cancer therapy, which is defined as a platelet count of <100×10^3^/μl (1). Myelosuppression leading to hematopoietic abnormalities induced by chemotherapy is the major cause; immune thrombocytopenia may also occur (2). Many reports indicated that patients with autoimmune diseases were more likely to develop immune thrombocytopenia induced by drugs (3, 4). However, since thrombocytopenia is a condition with many other inducements, the diagnosis of drug-induced immune thrombocytopenia (DITP) can easily be overlooked (5). Here, we report a case of DITP with bevacizumab as the most likely culprit drug in an ovarian cancer patient with a history of mixed connective tissue disease (MCTD). We also review existing literatures on immune thrombocytopenia induced by bevacizumab and discuss related mechanisms. Autoimmune predisposition as a trigger for DITP is also presented.

## Case report

**Figure 1:**
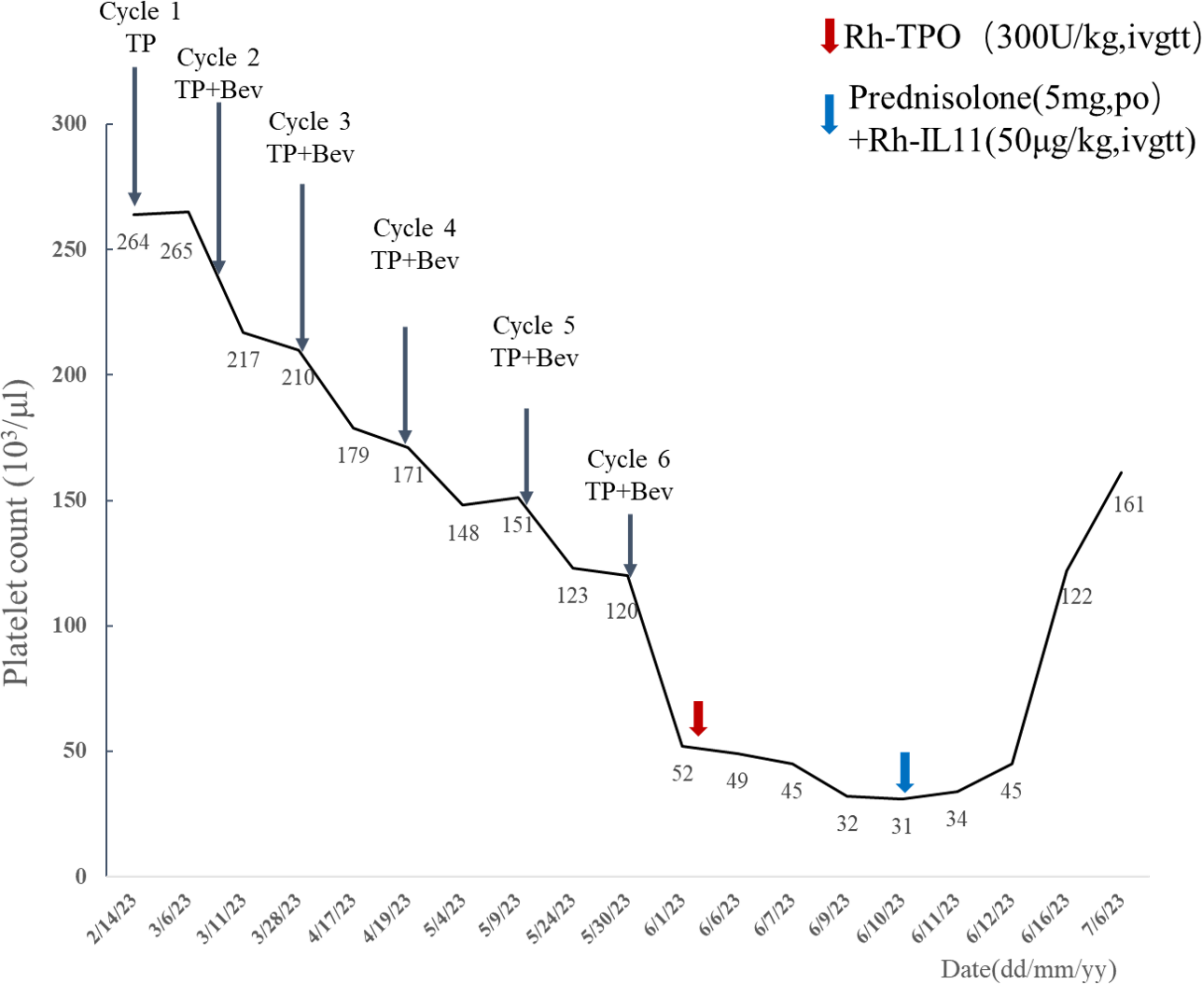
Changes in platelet count in relation to treatment.

In February 2023, a 68-year-old woman was diagnosed with stage IV high-grade serous ovarian carcinoma. The patient had a past medical history of MCTD presented with Raynaud’s phenomenon with fingers cold and white in winter, which was well controlled with oral prednisone (5 mg per 24 h), hydroxychloroquine (0.1 g, per 12 h), and aspirin (100 mg per 24 h) from November to February of the following year. Considering peritoneal metastasis, the patient received intravenous paclitaxel (135mg/m^2^) plus intraperitoneal cisplatin (75 mg/m^2^) (TP regimen) for day 1 every 3 weeks for six cycles after cytoreductive surgery. Bevacizumab (15 mg/kg) was administered intravenously at day 2 starting from cycle 2. With no other hematological abnormality found, the patient exhibited a gradual drift downward in platelet count within normal limits (**Figure 1**). Furthermore, the patient developed acute thrombocytopenia on the first day after the sixth cycle of bevacizumab infusion. Complete blood count showed an isolated decrease in platelet count from 120 × 10^3^/μl (as measured before chemotherapy infusion) to 52 × 10^3^/μl (**Figure 1**). Other hematological parameters, such as leukocyte (4.27 × 10^3^/μl) and neutrophil count (3.36 × 10^3^/μl) were in the normal range, and no liver function or coagulation disorders were displayed. She subsequently received recombinant human thrombopoietin (Rh-TPO), 300 U/kg intravenously. Despite this treatment, platelet count continued to drop to 31 × 10^3^/μl on day 10 after the administration of bevacizumab (**Figure 1**). The patient showed no signs of progression or metastases of malignancies with the unremarkable hypogastrium MRI and no elevation in biomarkers of cancers including HE4, CA125, and ROMA algorithm (**Table 1**). A bone marrow biopsy was disregarded by the patient due to its invasive nature and the absence of hemorrhagic complications. The patient had no fever or Raynaud’s syndrome signs. Autoimmunology parameters were conducted, and tests were negative except for ANA and anti-nRNP, which were similar to the tests before cancer therapy (**Table 1**). Anti-platelet antibody testing of the peripheral blood samples showed the presence of glycoprotein IIb (GPIIb) antibodies with flow fluorescence microsphere method. After excluding malignancy, myelosuppression, infection, pseudo-thrombocytopenia (PTCP), autoimmune diseases, or other drug-induced thrombocytopenia, DITP was finally diagnosed. According to Naranjo’s algorithm, bevacizumab was deemed the most likely drug with a score of 6 (probable) (see **Supplementary Table S1**). Therefore, treatment with oral prednisone (5 mg per 24 h) and intravenous recombinant human interleukin-11 (RhIL-11), 25 μg/kg, was started. Platelet count gradually increased up to 122 × 10^3^/μl 6 days later (**Figure 1**). After 42 days of the infusion, re-evaluation of autoimmunity parameters and platelet count was conducted, and changes in tests were unremarkable except for GPIIb, which was negative. The patient remained stable and was rechecked monthly, with no decrease in platelet count observed.

**Table 1:** Autoimmunity findings, platelet count, and biological markers of cancers prior to cancer therapy (baselines) and on day 10 and 42 after administration of bevacizumab (cycle 6).

Parameter	Baseline	Day 10	Day 42	Reference range
PLT, ×10^3^/μl	264	31	142	125–350
CRP, mg/l	21.60	53.14	<3.22	<8.2
ESA, mm/h		59	34	0–20
IgG, mg/dl		11.9	17.3	8.6–17.4
IgA, mg/dl		2.2	2.85	1.0–4.2
IgM, mg/dl		0.718	0.96	0.5–2.8
ASO, IU/ml		73	9.19	<408
C3, mg/dL		1.350	1.180	0.7–1.4
ANA	+	+	+	negative
ANCA	−	−	−	negative
ACA	−	−	−	negative
Anti-RNP	+	+	+	negative
Anti-dsDNA	−	−	−	negative
Anti-GP IIb		+	−	negative
Anti-GP IIa		−	−	negative
Anti-GP Ib		−	−	negative
Anti-GP IX		−	−	negative
Anti-GMP140		−	−	negative
CA125, U/ml	2419	24.3	29.0	<35
CA199, U/ml	3.5	8.27	7.29	<27
HE4, pmol/L	1366	118	124	<82.9
ROMA	99.4	31.2	35.2	<29.9%(Postmenopausal)

PLT, platelet; CRP, C-reactive protein; ESA, erythrocyte sedimentation rate; Ig, immunoglobulin; ASO: anti streptolysin C, complement; ANA, antinuclear antibodies; ANCA, anti-neutrophil antibodies; ACA, anticardiolipin antibodies; anti-RNP, antinuclear ribonucleoprotein; ds-DNA, double-stranded deoxyribonucleic acid; GP, P-glycoprotein; HE, human epididymis protein; ROMA, Risk ovarian malignancy algorithm; +, positive; −, negative.

## Discussion

Possible causes of thrombocytopenia in the present patient included cancer-therapy-related thrombocytopenia, exacerbation of MCTD, primary immune thrombocytopenia (ITP), and PTCP. Thrombocytopenia maybe caused by cancer directly with tumor involvement of bone marrow and spleen, which occurred most often in patients with known metastatic cancer and exacerbation of the disease (6, 7). It was ruled out that the thrombocytopenia was directly caused by the ovarian cancer or cancer-associated factors with unremarkable biomarkers and MRI results. Severe thrombocytopenia was a rare risk of paclitaxel plus cisplatin and bevacizumab in ovarian cancer, occurring in up to 2%–4% of patients as reported in clinical trials (8, 9). Thrombocytopenia due to myelosuppression is typically accompanied by leukopenia (10). In this case, the platelet count decreased progressively and showed acute isolated thrombocytopenia after several cycles, which was similar to the gradual thrombocytopenia induced by long-term exposure to trastuzumab (11). Although thrombocytopenia occurred in MCTD, the frequency was rare, as most were case reports, and thrombocytopenia was usually correlated with positive ACA (12). Thrombocytopenia secondary to MCTD was not present in the patient, as there were no clinical features of Raynaud’s syndrome and autoimmunity parameters showing unremarkable changes. ITP was also excluded as well since thrombocytopenia promptly recovered within 16 days after the discontinuation of bevacizumab, and this clinical manifestation is not consistent with ITP. PTCP was ruled out, as no platelet aggregation was observed in a peripheral blood smear (13). Then, clinical and laboratory criteria for establishing a causative relationship between a drug and thrombocytopenia were used, and possible score was found (5, 14). In the present case, drug exposure precedes thrombocytopenia, and recovery from thrombocytopenia was complete and sustained after drugs withdrawal. Other etiological factors of thrombocytopenia were excluded. Serum antiplatelet antibody testing revealed antibodies against GPIIb.

In the context of advanced cancer therapy, distinguishing the specific drug causing thrombocytopenia in patients receiving many medication treatments simultaneously is a challenging issue (15). Several elements were provided to analyze that acute thrombocytopenia in the current patient was probably triggered by bevacizumab. 1) Neither cases of cisplatin and paclitaxel have been reported nor have they been documented in the adverse reaction systems. Bevacizumab associated with thrombocytopenia has been reported in several cases, which was considered to be related to immunity, and the French PharmacoVigilance Database showed relevant records. 2) The administration’s relationship in time to the onset of clinical manifestations was clear. 3) Recovery from thrombocytopenia did not occur rapidly after withdrawal of bevacizumab. The presence of antiplatelet antibodies against GPIIb, which may be a chronic effect of immune thrombocytopenia caused by bevacizumab (a drug with a half-life of 10 days) could be the reason for this. It was possible that the absence of GPIIb in a later period and the subsequent recovery after corticosteroid treatment could be attributed to this. 4) The probable score of the relationship between the drug and development of thrombocytopenia (Naranjo scale = 6) was found with Naranjo’s algorithm (11).

Immune-mediated thrombocytopenia is a rare complication of bevacizumab that had only few reports in the literature about glioma, colon cancer, and macular degeneration, which was an off-label use. Glioma cells were reported to have the ability to evade the immune system and foster an immunosuppressive milieu by various mechanisms, notably by diminishing the recruitment of immune cells, secreting immunosuppressive cytokines, and activating the STAT3 pathway. The STAT3 pathway facilitated the synthesis of vascular endothelial growth factor (VEGF), which was the target of bevacizumab (16). VEGF was often overexpressed in epithelial ovarian cancer and therefore an attractive target for therapy (17). The reported cases of malignancies all received many prior therapies with resulting decreased bone marrow reserve (see **Table 2**). Our patient received five cycles of bevacizumab and paclitaxel-cisplatin chemotherapy before. The clinical features of bevacizumab-induced thrombocytopenia varied. Both were diagnosed with colon cancer; the case of bevacizumab-induced thrombocytopenia reported by Leal et al. had no bleeding with mild thrombocytopenia (18). In the other case reported by Kumar et al., the patient developed severe thrombocytopenia with melena and epistaxis, attributable to bevacizumab (19). Our patient had no bleeding, and platelet count was slightly decreased. Although it was not widely adapted in the management of thrombocytopenia in cancer patients, a bone marrow biopsy was a favorable method to confirm the immune thrombocytopenia especially in the cases where the diagnosis remained uncertain (21, 22). Immune-related thrombocytopenia induced by bevacizumab was usually reversible with the treatment with corticosteroids and thrombopoietic growth factors, such as Rh-TPO or Rh-IL11. Under certain circumstances, mild thrombocytopenia may not require specific treatment.

**Table 2:** Known cases of bevacizumab-mediated immune thrombocytopenia.

Study	Publication Year	Age	Gender	Disease	Lowest PLT (×10^3^/μl)	Treatment	Concurrent Manifestation	Antiplatelet Antibodies	Bone Marrow Examination	Treatments Prior to DITP	Outcome
Leal, T. et al. (18)	2010	36	Female	Recurrent high-grade glioma	55	None	None	ND	ND	CPT-11; temozolomide; erlotinib; CCI779; TMZ; tamoxifen; radiotherapy; bevacizumab	Recovered
Kumar, J. et al. (19)	2012	59	Male	Relapsed adenocarcinoma of colon	6	Platelet apheresis;	Melena, epistaxis	ND	Increased megakaryocytes	FOLFOX-4; FOLFIRI; bevacizumab	Recovered
Ozaslan, E. et al. (20)	2015	68	Female	Metastatic colon cancer	53	Methylprednisolone; Hydroxychloroquine; Aspirin	Drug induced lupus erythematosus	ND	ND	Capecitabine; oxaliplatin; bevacizumab	Recovered
Li, T. et al (21)	2017	77	Male	Right-sided macular degeneration	3	Apheresis platelet; examethasone; IVIG; rituximab; romiplostim	Subconjunctival hemorrhage and proptosis, epistaxis, hematuria, gingival bleeding; extensive extremity ecchymosis	GPIV, HLA	Megakaryocytic hyperplasia	None	Recovered
Pasquariello, S. et al. (4)	2023	49	Female	Rectal adenocarcinoma	2	Blood transfusion dexamethasone	Purpura	ND	ND	FOLFOX-4; FOLFOX6; panitumumab; bevacizumab	Recovered
The present case	2023	68	Female	Ovarian carcinoma	31	Rh-TPO; prednisone; IL-11	None	GP IIb	ND	Cisplatin; paclitaxel; bevacizumab	Recovered

ND, not detected; CPT-11, irinotecan; CCI779, temsirolimus; TMZ, temozolomide; IVIG, intravenous immunoglobulin; FOLFOX, folinic acid, 5-fluorouracil, and oxaliplatin FOLFIRI, folinic acid, 5-fluorouracil, and irinotecan; HLA, human leukocyte antigen; rh-TPO, recombinant human thrombopoietin; IL-11, interleukin-11.

Bevacizumab is a highly effective humanized monoclonal antibody against VEGF that disrupts the blood supply to the tumor, making it an essential treatment option for cancer patients (23). Albeit the exact mechanisms that lead to these side effects are not well understood, increasing evidence represented the immunological characteristics of bevacizumab (23, 24). It has been concluded that bevacizumab was concentrated in platelets and formed immune complexes with VEGF *in vitro* and *in vivo (* 25, 26). Meyer et al. and Nomura et al. (24, 27) described that the bevacizumab associated immune complexes activated platelets via the IgG receptor FcγRIIa and directly caused thrombocyte aggregation, which was similar to the mechanism of heparin-induced thrombocytopenia (HIT), which was typically an immune-mediated thrombocytopenia (28).

The effects of autoimmune disorders and their corresponding therapeutic medications on individuals are complex and fascinating. Some of the immunosuppressive agents used to treat autoimmune diseases may directly or indirectly be associated with the subsequent development of malignancies (29). The autoimmune status of patients should also be considered in the setting of drug-mediated immune-like complexes as a triggering factor for immune thrombocytopenia. Patients diagnosed with HIT have a high incidence of comorbid autoimmune diseases, namely, systemic lupus erythematosus, rheumatoid arthritis, and Hashimoto’s thyroiditis, with age being a significant variable (30, 31, 32). In the case reported by Pasquariello et al., the patient with colorectal cancer developed sever thrombocytopenia when treated with panitumumab or bevacizumab, whose immunology tests of anti-nucleus antibody was positive (4). According to cases reported in the French PharmacoVigilance database, 25% of patients with ITP induced by serotonin reuptake inhibitors or bevacizumab had autoimmune antecedents (33). In another case, the patient with colorectal cancer developed methylprednisolone-induced thrombocytopenia after the oxaliplatin-induced thrombocytopenia, attributable to the oxaliplatin immune-induced syndrome owing to anti-GPIIbIIIa and anti-CD36 antibodies (34). Drugs including antineoplastic drug-related immune thrombocytopenia may be more likely to occur in patients with autoimmune diseases or an autoimmune activated state. More research will be needed to support this idea.

Here, we report the first case of an ovarian cancer patient with mixed connective tissue disease who developed an immune-related thrombocytopenia during chemotherapy. However, due to the rarity of this clinical occurrence and the scarcity of existing literature, it is challenging to comprehensively document the event and fully confirm the correct diagnostic and therapeutic approach. Our case highlights an attention on bevacizumab-induced immune thrombocytopenia. In addition, the current case report provides clinical data relevant to the largely unexplored question of DITP in patients with a pre-existing autoimmune state.

## Data availability statement

The original contributions presented in the study are included in the article/**Supplementary Material**. Further inquiries can be directed to the corresponding authors.

## Ethics statement

Written informed consent was obtained from the individual(s) for the publication of any potentially identifiable images or data included in this article.

## Author contributions

YZ: Writing – original draft, Writing – review & editing, Data curation, Formal analysis, Project administration. FY: Writing – review & editing, Investigation, Project administration. JW: Data curation, Writing – review & editing. HF: Data curation, Funding acquisition, Writing – review & editing. FS: Formal analysis, Supervision, Writing – review & editing. JL: Formal analysis, Writing – review & editing. DL: Formal analysis, Project administration, Writing – review & editing.
