# Supplementary material for: Bevacizumab-induced immune thrombocytopenia in an ovarian cancer patient with mixed connective tissue disease: case report and literature review
Source: Front Immunol. 2024 Jun 5;15:1382964. doi: 10.3389/fimmu.2024.1382964 (PMC11188369; doi:10.3389/fimmu.2024.1382964)
Supplement: Supplementary file 1 [file Table_1.pdf]

Supplementary Material

NO.	Question	Yes	No	Unknow
1	Were there any prior documented reports of unfavorable reactions (side effects) to the drug?	+1	0	0
2	Did the adverse event (side effect) following drug appear after the drug was injected?	+2	-1	0
3	Did the adverse event (side effect) improve when the drug withdrawn, or a specific antagonist was used?	+1	0	0
4	Are there any other possible causes (other than the drug) that could have caused the reaction?	-1	+2	0
5	When the drug was re-administered, did the reaction return?	+2	-1	0
6	Did the reaction reappear after the placebo was administered?	-1	+1	0
7	Was the drug found in blood (or other bodily fluids) at toxic concentrations?	+1	0	0
8	Did the adverse event (side effect) brought on by the drug get worse as the dose was increased? Or did the unpleasant event (side effect) that followed the drug get milder as the dose was reduced?	+1	0	0
9	Did you have a similar adverse event (side effect) following the drug to the same or similar drug in any previous exposure?	+1	0	0
10	Was there any verifiable evidence to support the adverse event (side effect) following the drug?	+1	0	0
Total score		6		

Supplementary Table 1. The relationship of immune-related thrombocytopenia onset of bevacizumab according to Naranjo Algorithm. The definition of "probable" was found with a score of 6. "Definite" (score 9), "Probable" (score between 5 and 8), "Possible" (score between 1 and 4), and "Doubtful" (score 0).
